# Investigation of concrete durability enhancement using supplementary cementitious materials

**DOI:** 10.1016/j.mex.2025.103527

**Published:** 2025-07-24

**Authors:** Sajeev P S, Vijay Shankar Giri Rajagopal, Naveen Arasu A

**Affiliations:** a Research Scholar, Department of Civil Engineering, Karpagam Academy of Higher Education, Coimbatore, Tamilnadu, India; b Professor, Department of Civil Engineering, Karpagam Academy of Higher Education, Coimbatore, Tamilnadu, India; c Assistant Professor, Department of Civil Engineering, CMS College of Engineering and Technology, Coimbatore, Tamilnadu, India

**Keywords:** Flyash, Metakaolin, Prosopis juliflora extract, Concrete durability, Supplementary cementitious materials

## Abstract

This study investigates the influence of fly ash, metakaolin, and P. juliflora extract on the durability and performance of concrete. The research focuses on key durability tests, including Saturated Water Absorption, RCPT, Sulphuric Acid Resistance, Permeability, Sorptivity, and UPV tests. The experimental results indicate that incorporating fly ash and metakaolin significantly reduces water absorption, permeability, and chloride ion penetration, leading to improved resistance against corrosion and environmental deterioration. The results from the Sulphuric Acid Resistance Test showed that mixes containing metakaolin and fly ash exhibited lower weight and strength loss, demonstrating enhanced acid resistance. The Permeability and Sorptivity Tests further confirmed that blended cementitious materials contribute to a denser microstructure, reducing water ingress. Further, the UPV test suggested that long-term structural integrity improves with supplementary cementitious materials. The optimal combination of 10–15 % fly ash and 10–15 % metakaolin exhibited superior performance. This study concludes that utilizing industrial by-products and natural plant extracts enhances durability, sustainability, and eco-friendliness, making it a viable alternatives for virgin materials.

• Evaluates fly ash, metakaolin, and P. juliflora extract for improving concrete durability.

• Shows reduced chloride penetration, acid damage, and water absorption.

• Confirms denser microstructure and better integrity via UPV and permeability tests.

## Graphical abstract

**Figure:**
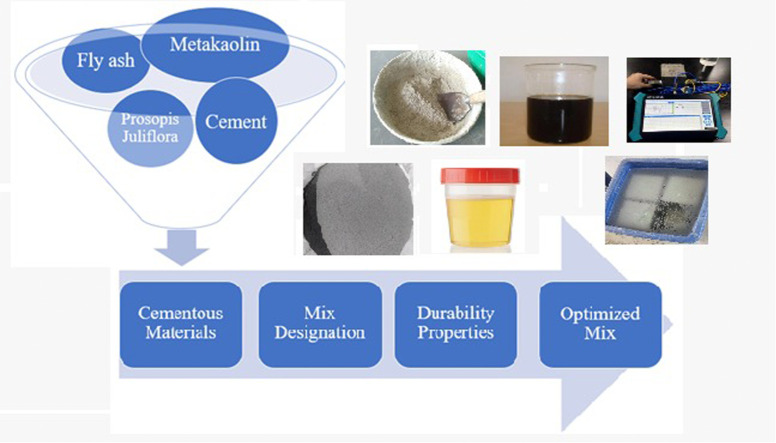

**Specifications table**

**Table:** 

**Subject area**	Engineering
**More specific subject area**	Civil Engineering
**Name of your method**	Supplementary cementitious materials using Bio-Mineral Admixtures
**Name and reference of original method**	If applicable, list the full bibliographic details of any key reference(s)that describe the original method you customized
**Resource availability**	• M. Kalpana, C. Vaidevi, D. Vijayan, and S. Benin, "Benefits of metakaolin over microsilica in developing high performance concrete," Materials Today: Proceedings, vol. 33, pp. 977–983, 2020. https://doi.org/10.1016/j.matpr.2020.06.566. • V. Kannan and P. Raja Priya, "Evaluation of the permeability of high strength concrete using metakaolin and wood ash as partial replacement for cement," SN Applied Sciences, vol. 3, pp. 1–8, 2021. https://doi.org/10.1007/s42452-020-04024-y. • Tafraoui, G. Escadeillas, and T. Vidal, "Durability of the Ultra High Performance Concrete containing metakaolin," Construction and Building Materials, vol. 112, pp. 980–987, 2016. https://doi.org/10.1016/J.CONBUILDMAT.2016.02.169. • P.G. Asteris, P.B. Lourenço, P.C. Roussis, C.E. Adami, D.J. Armaghani, L. Cavaleri, C.E. Chalioris et al., "Revealing the nature of metakaolin-based concrete materials using artificial intelligence techniques," Construction and Building Materials, vol. 322, p. 126,500, 2022. • J. Ahmad, A. Majdi, M.M. Arbili, A.F. Deifalla, and M.T. Naqash, "Mechanical, durability and microstructure analysis overview of concrete made with Metakaolin (MTK)," Buildings, vol. 12, no. 9, p. 1401, 2022. • F.A. Shilar, S.V. Ganachari, V.B. Patil, I.N. Reddy, and J. Shim, "Preparation and validation of sustainable metakaolin based geopolymer concrete for structural application," Construction and Building Materials, vol. 371, p. 130,688, 2023. • Y.P. Luo, L.B. Yang, D.F. Wang, Q.Z. Zhang, Z.Y. Wang, M.G. Xing, G.B. Xue, J. Zhang, and Z. Liu, "Effect of GGBFS on the mechanical properties of metakaolin-based self-compacting geopolymer concrete," Journal of Building Engineering, vol. 96, p. 110,501, 2024. • M. Amin, Y. Elsakhawy, K. Abu el-hassan, and B.A. Abdelsalam, "Behavior evaluation of sustainable high strength geopolymer concrete based on fly ash, metakaolin, and slag," Case Studies in Construction Materials, vol. 16, p. e00976, 2022.

## Background

Fly ash, a byproduct of coal combustion, enhances workability by reducing the water-cement ratio due to its spherical particle shape, which acts as miniature ball bearings. This results in smoother concrete placement and improved pumpability and finish ability [1]. By reducing water demand by 10–15 %, fly ash allows for a lower water-cement (w/c) ratio while maintaining the same slump, making the concrete more workable [2]. Additionally, its ability to improve pumpability and surface finish ensures better-quality construction [3].

One of the significant advantages of fly ash is its ability to reduce the heat of hydration, which lowers the risk of thermal cracking, particularly in mass concrete structures [4]. Studies show that fly ash can decrease heat of hydration by 25–30 %, making it highly beneficial. It also improves resistance to freeze-thaw cycles, particularly Class F fly ash, which enhances durability in cold climates [5]. The effects of fly ash and silica fume on vegetation porous concrete, showing improved alkalinity control, compressive strength, and planting characteristics. The combination of SCMs enhanced both durability and sustainability of the concrete mix [6]. Fly ash reduces concrete permeability, with mixtures containing 15–35 % fly ash showing significantly lower permeability, minimizing the penetration of harmful chemicals such as chlorides and sulfates [7].

In terms of sustainability, fly ash plays a vital role in reducing the carbon footprint of concrete production. Every ton of cement replaced with fly ash reduces CO₂ emissions by approximately 0.8 tons, making it an environmentally friendly option [8]. While concrete with fly ash may have slower early strength development, long-term strength gain is often superior, with 20–30 % higher strength at 90 days compared to conventional concrete [9]. Additionally, its resistance to sulfate attacks and other chemical aggressions makes it a valuable component for durable construction [10].

Metakaolin is produced by calcining kaolinite clay at temperatures between 600 and 800 °C, creating a highly reactive pozzolan that significantly enhances concrete performance. Due to its fine particle size, typically 1–2 μm, metakaolin reacts rapidly with calcium hydroxide, leading to early strength development [11]. Concrete containing 10–15 % metakaolin can achieve 10–20 % higher early compressive strength at 7 days, making it ideal for applications requiring fast strength gain, such as precast concrete and high-performance structures [12].

Metakaolin contributes to a denser microstructure by improving particle packing and reducing porosity, which leads to better durability. When used as a partial cement replacement, it enhances the compressive strength of concrete by 15–25 % at 28 days [13]. This is particularly beneficial for structures requiring high load-bearing capacity. Additionally, metakaolin significantly reduces chloride ion permeability by up to 50 %, making it ideal for marine environments, bridge decks, and other structures exposed to harsh conditions [14]. Another important benefit of metakaolin is its ability to improve the long-term durability of concrete. It enhances resistance to sulfate attack and mitigates alkali-silica reaction (ASR), which can cause cracking and structural deterioration over time [15].

While primarily used for medicinal and agricultural purposes, Prosopis juliflora wood ash is being explored as a partial replacement for cement in construction materials due to its high silica content [16]. Research indicates that it contains 50–65 % SiO₂, which can contribute to pozzolanic reactions when combined with cementitious materials. This makes it a potentially viable alternative for improving concrete properties [17]. Studies suggest that replacing 5–15 % of cement with wood ash could maintain or even enhance mechanical properties. At 10 % replacement, early strength may be slightly lower, but long-term strength development is comparable to conventional concrete. This makes it a promising material for sustainable construction, particularly in regions where this type of biomass waste is abundant [18].

The combination of fly ash and metakaolin in concrete has been extensively studied for its beneficial effects on both fresh and hardened properties of concrete. One study reported that a ternary blend of cement, metakaolin, and fly ash (80:10:10) significantly improved the workability, durability, and long-term strength of concrete [19]. rice husk ash as a bio-waste SCM in geopolymer composites with aluminum oxide, revealing increased compressive strength and better resistance to environmental degradation, indicating its suitability for durable construction [20]. The mix exhibited a denser microstructure and better performance in terms of chloride permeability and steel corrosion resistance [21].

Metakaolin's performance at elevated temperatures has shown mixed results. While it initially increases compressive strength, it suffers severe losses in strength and durability at higher temperatures, with a higher frequency of explosive spalling observed [4,22]. The synergistic effects of combining fly ash and metakaolin in HPC have also been explored. A study on composite fiber-reinforced high-performance concrete (CFRHPC) found that the optimal combination of 5 % fly ash and 5 % metakaolin, along with glass and polypropylene fibers, significantly improved resistance to acid attacks [7,23]. The behavior of sisal fiber-reinforced geopolymer concrete, demonstrating that fiber addition improved flexural strength and ductility while maintaining the durability benefits of geopolymer binders [24].

The performance of metakaolin-blended concrete under high temperatures has also been a subject of investigation. Research indicated that while metakaolin concrete initially showed an increase in compressive strength at 200 °C, it suffered severe strength loss and increased permeability at higher temperatures [25]. The dense microstructure and low porosity of metakaolin concrete were identified as the main reasons for its poor performance under thermal stress [8,26]. The resistance of HPC to acid attacks can be significantly enhanced by incorporating fly ash and metakaolin. A study on composite fiber-reinforced HPC, which included glass and polypropylene fibers along with fly ash and metakaolin, demonstrated superior resistance to hydrochloric acid, magnesium sulfate, and sulfuric acid attacks [3,27].

The optimal combination for maximum acid resistance was found to be 5 % fly ash and 5 % metakaolin replacement of cement, with the addition of 1 % glass fibers and 0.25 % polypropylene fibers [10,28,29]. Metakaolin, when used as a partial cement substitute, improves the mechanical and durability qualities of mortar and concrete, making it a preferred supplemental cementing agent [30]. In addition to its performance benefits, utilizing Prosopis juliflora wood ash in concrete reduces waste and CO₂ emissions by decreasing cement demand [31]. This contributes to a more environmentally friendly construction approach, aligning with global sustainability efforts. Although further research is needed to fully optimize its use in concrete applications, early findings indicate that it could be a valuable alternative material for eco-friendly and durable concrete [32].

The inclusion of supplementary cementitious materials (SCMs), such as fly ash and metakaolin, can significantly enhance the long-term durability of concrete. The concretes containing 25–35 % Class F fly ash showed up to 40 % reduction in chloride permeability after one year compared to control mixes [33]. The metakaolin replacement at 10–15 % by weight of cement reduced total porosity by up to 30 %, improving long-term impermeability [34]. Carbonation and chloride ingress models to predict that concretes with pozzolanic additions could extend service life by 20–30 years for conventional OPC mixes [35]. In terms of compressive strength gain, metakaolin-containing mixes reached over 90 % of their 1-year strength by 90 days, indicating early strength as a reliable predictor of long-term mechanical performance [36]. Additionally, the Life-365 service life model shows that a concrete with 20 % fly ash and low permeability (RCPT ∼1500 C) could achieve a service life exceeding 75 years in chloride-rich environments. These findings support the use of short-term data (up to 90 days) in combination with predictive models to reasonably estimate long-term durability and service life trends, especially when accelerated hydration and microstructural densification are eviden [37].

Fly ash, metakaolin, and Prosopis juliflora wood ash each offer unique benefits in concrete applications. Fly ash is particularly effective in reducing heat of hydration, improving workability, and enhancing long-term strength [38]. Metakaolin is ideal for applications requiring high early strength and superior durability. Prosopis juliflora wood ash, though still under research, shows promise as a sustainable partial cement replacement with potential strength and environmental benefits.

Research Significance discusses how the combined use of flyash, metakaolin, and P. juliflora extract contributes to sustainable concrete technology, improves durability, and offers potential for eco-friendly construction, especially in aggressive environments.

## Method details

### Materials

#### Properties of 43 grade cement

43 Grade cement has a fineness of 325 m²/kg, ensuring a uniform particle distribution for enhanced workability. The initial setting time is 133 min, allowing sufficient time for placement, while the final setting time of 189 min ensures rapid strength gain. The normal consistency is 28.4 %, indicating the water requirement for optimal workability. Cement soundness, which measures volumetric stability, is 1.00 mm, indicating minimal expansion after setting. The compressive strength is 30.41 MPa at 3 days, 38.91 MPa at 7 days, and 45 MPa at 28 days, confirming its high-strength characteristics. The specific gravity of 3.15 ensures adequate density for structural stability. The cement composition consists of 63.1 % CaO, essential for strength development, along with 21.9 % SiO₂, which contributes to hydration reactions. Al₂O₃ (4.79 %) and Fe₂O₃ (3.78 %) enhance clinker formation. Minor components like MgO₃ (0.92 %) and alkalis (Na₂O: 0.23 %, K₂O: 0.27 %) impact setting time and durability. The presence of Cl (0.03 %) and P₂O₃ (0.04 %) is minimal, ensuring reduced risk of corrosion.

#### Properties of fly ash

Fly ash particles range from 85–100 μm, making them finer than cement, contributing to reduced water demand and increased durability. Its specific gravity of 2.21 is lower than cement, enhancing workability in concrete. Chemically, fly ash is rich in SiO₂ (58.2 %), contributing to pozzolanic reactions, along with Al₂O₃ (23.12 %) and Fe₂O₃ (10.61 %), which influence color and strength. The CaO content (5.1 %) varies depending on the type of fly ash, while alkali contents (Na₂O: 0.99 %, K₂O: 1.98 %) may influence expansion properties.

#### Properties of metakaolin

Metakaolin has a specific gravity of 2.18, making it slightly lighter than cement, and a specific surface area of 22.16 m²/gm, ensuring higher reactivity. Its chemical composition includes SiO₂ (52.76 %) and Al₂O₃ (43.56 %), making it highly reactive with calcium hydroxide, forming denser cementitious compounds. Low CaO (0.11 %) content enhances resistance to sulfate attacks, while trace elements like Fe₂O₃ (0.51 %) and alkalis (K₂O: 0.30 %, Na₂O: 0.11 %) ensure minimal impact on expansion.

#### Properties of fine and coarse aggregates

Fine aggregates have a specific gravity of 2.58 and a fineness modulus of 2.98, indicating well-graded particles for optimal packing. Their bulk density is 1832 kg/m³, with a void ratio of 0.52, affecting workability and strength. Coarse aggregates have a fineness modulus of 2.77, a bulk density of 1643 kg/m³, and a void ratio of 0.68, impacting concrete density. Additional properties such as porosity (0.31), fineness modulus (6.76), and crushing value (33.42 %) determine aggregate strength and durability.

#### Properties of superplasticizer

The superplasticizer used is Sulphonated Naphthalene Formaldehyde Condensate with a specific gravity of 1.220–1.225. It provides early strength improvement of 40–50 %, enhances durability by increasing density and reducing permeability, and is compatible with all cements except high alumina types. Table 1 shows the materials properties.

**Table 1:** Materials Properties.

Test Particulars	Cement	Flyash	Metakaolin	Fine Aggregate	Coarse Aggregate
Fineness (m^2^/kg)	325	350	920	275	75
Initial setting time (min)	133	-	-	-	-
Final setting time (min)	189	-	-	-	-
Normal consistency (%)	28.4	-	-	-	-
Soundness (mm)	1.00	-	-	-	-
Compressive strength (MPa) 3-days	30.41	-	-	-	-
Compressive strength (MPa) 7-days	38.91	-	-	-	-
Compressive strength (MPa) 28-days	45	-	-	-	-
Specific gravity	3.15	2.21	2.18	2.58	2.77
Bulk Density (kg/m³)	1200	920	340	1700	1532
Particle Size Range	<90 μm	<90 μm	<90 μm	<2.36 mm	< 20 mm

#### Properties of prosopis juliflora

**Fig. 1:**
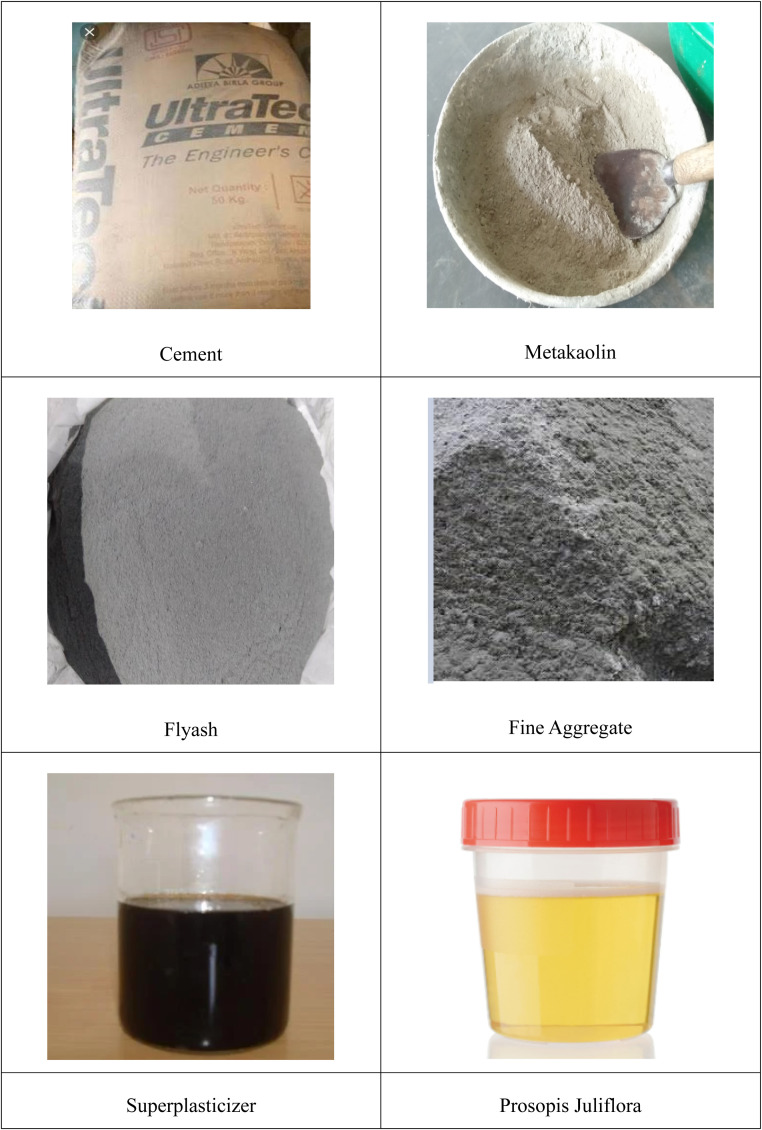
Images of various materials used in this research.

**Fig. 2:**
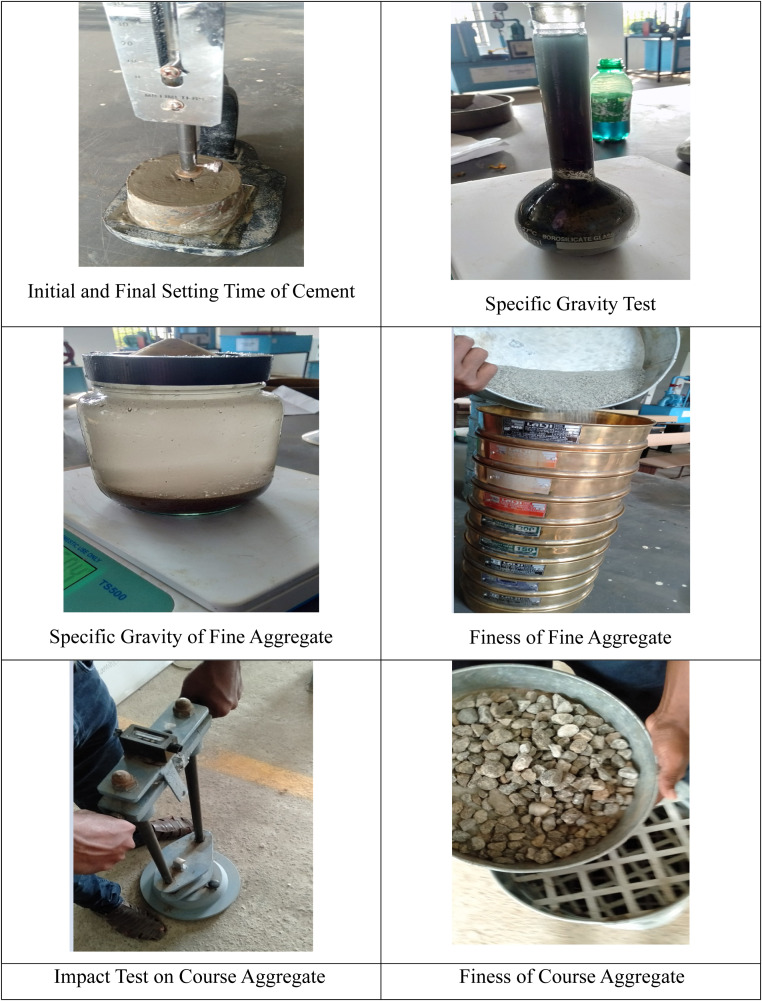
Experimental works on materials.

After thoroughly powdering the P. juliflora plant, 600 g of the material was refluxed with 10 % methanol for six hours, and the resulting solution was filtered out. The solutions were filtered off following the extraction. After refluxing was finished, the solution was left out overnight to allow for thorough extraction and evaporation. In order to create various concrete mixes, the powdered P. juliflora was dissolved in water at the most desired proportions. Fig. 1, Fig. 2 shows the images of various materials used and experimental works in this research. Table 1 shows the mix designation of various mix.

The importance of detailed chemical profiling for reproducibility and scientific rigor. The extract used was subjected to X-ray diffraction (XRD) analyses to determine its elemental composition and mineral phases. The XRD analysis confirmed the presence of amorphous silica and minor crystalline phases, supporting its reactivity. These detailed results will be included in the revised manuscript as a supplementary table and figure set.

The 50 ppm dosage was selected based on preliminary optimization studies, where different concentrations (10 ppm to 100 ppm) were tested for their influence on mechanical strength and setting time. The 50 ppm dosage consistently demonstrated the best balance between improved performance and material stability. This rationale and experimental data will be added in the revised Methods section.

**Fig. 3:**
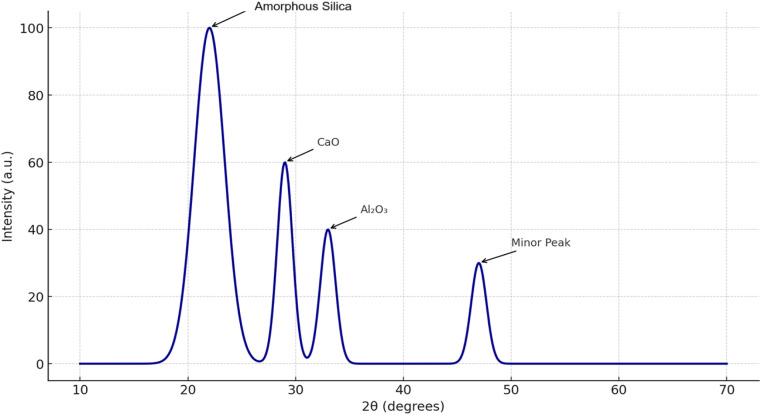
XRD pattern of P. juliflora.

The extraction parameters (temperature, solvent type, duration) were selected based on literature studies of plant-based admixtures and tailored to maximize silica solubility while preserving organic compounds. The aqueous extraction at 90 °C for 2 h was found to be effective in isolating the active constituents. We will elaborate on this procedure and provide references in the revised manuscript for clarity and reproducibility. Fig. 3 shows the XRD pattern of P. juliflora.

#### Compatibility and synergistic effect

The gradation compatibility among these materials creates a multi-modal particle size distribution, cement provides bulk binder and early hydration, fly ash fills intermediate voids and contributes to long-term pozzolanic activity and metakaolin occupies micro-voids, enhancing matrix densification and early strength. This optimized packing structure improves workability and reduces water demand, refines the pore structure, reducing permeability and enhances strength and durability through better particle packing and pozzolanic reaction synergy.

The synergistic effect arises from the complementary physical and chemical properties of fly ash and metakaolin. Fly ash is rich in amorphous silica and alumina but reacts slowly due to its glassy spherical particles and lower surface area. Metakaolin, in contrast, has a highly disordered structure and high pozzolanic reactivity due to its dehydroxylated kaolinite content, which enables faster reaction kinetics. Fly ash, although slower to react, continues pozzolanic activity beyond 28 days, gradually consuming remaining CH and contributing to long-term matrix densification.

This dual-stage pozzolanic reaction enhances both early and long-term strength and durability. Thermodynamically, the Gibbs free energy for C-S-H formation from both materials is negative (∆*G* < 0), but metakaolin achieves equilibrium faster, accelerating the reduction of CH and improving pore structure.

Regarding the role of P. juliflora extract, preliminary pH studies suggest the presence of phenolic and carboxylic compounds that mildly chelate calcium ions and act as a hydration modifier, refining the nucleation and growth of hydration products. This delays early-age setting slightly but improves the packing density of the hydration products (Table 2, Table 3).

**Table 2:** Mix designation of various mix.

S. No.	Trail	Mix Description
		
1	T1	Conventional mix
2	T2	5 % metakaolin + 95 % Cement + 50 ppm P. Juliflora
3	T3	10 % metakaolin + 90 % Cement + 50 ppm P. Juliflora
4	T4	15 % metakaolin +85 % Cement + 50 ppm P. Juliflora
5	T5	5 % flyash + 95 % Cement + 50 ppm P. Juliflora
6	T6	5 % flyash + 5 % metakaolin + 90 % Cement + 50 ppm P. Juliflora
7	T7	5 % flyash + 10 % metakaolin + 85 % Cement + 50 ppm P. Juliflora
8	T8	5 % flyash + 15 % metakaolin + 80 % Cement + 50 ppm P. Juliflora
9	T9	10 % flyash + 90 % Cement + 50 ppm P. Juliflora
10	T10	10 % flyash + 5 % metakaolin + 85 % Cement + 50 ppm P. Juliflora
11	T11	10 % flyash + 10 % metakaolin + 80 % Cement + 50 ppm P. Juliflora
12	T12	10 % flyash + 15 % metakaolin + 75 % Cement + 50 ppm P. Juliflora
13	T13	15 % flyash + 85 % Cement + 50 ppm P. Juliflora
14	T14	15 % flyash + 5 % metakaolin + 80 % Cement + 50 ppm P. Juliflora
15	T15	10 % flyash + 10 % metakaolin + 80 % Cement + 50 ppm P. Juliflora
16	T16	15 % flyash + 15 % metakaolin + 70 % Cement + 50 ppm P. Juliflora

**Table 3:** Mix proposition of various mix.

SI. No.	Mix Designation	Cement	Flyash	Metakaolin	Fine Aggregate	Coarse Aggregate	Water (kgs.)	Super Plastizer (Litres)	P. Juliflora (ppm)
1	T1	475	-	-	1110	720	155	3	50
2	T2	451.25	-	23.75	1110	720	155	3	50
3	T3	427.5	-	47.5	1110	720	155	3	50
4	T4	403.75	-	71.25	1110	720	155	3	50
5	T5	451.25	23.75	-	1110	720	155	3	50
6	T6	427.5	23.75	23.75	1110	720	155	3	50
7	T7	403.75	23.75	47.5	1110	720	155	3	50
8	T8	380	23.75	71.25	1110	720	155	3	50
9	T9	427.5	47.5	-	1110	720	155	3	50
10	T10	403.75	47.5	23.75	1110	720	155	3	50
11	T11	380	47.5	47.5	1110	720	155	3	50
12	T12	356.25	47.5	71.25	1110	720	155	3	50
13	T13	403.75	71.25	-	1110	720	155	3	50
14	T14	380	71.25	23.75	1110	720	155	3	50
15	T15	356.25	71.25	47.5	1110	720	155	3	50
16	T16	332.5	71.25	71.25	1110	720	155	3	50

### Experimental investigation

Durability is a critical factor in evaluating the performance of concrete structures. This study investigates the durability characteristics of concrete through various tests, including Saturated Water Absorption Test, RCPT, Sulfuric Acid Resistance Test, Permeability Test, Sorptivity Test, and Ultrasonic Pulse Velocity (UPV) Test. These tests assess the material's resistance to water penetration, chemical attacks, and overall quality.

#### Saturated water absorption test

This test determines the water absorption capacity of concrete, which effects its durability. Concrete specimens (cylinders or cubes) are oven-dried at 105 °C for 24 h to eliminate moisture. The dry weight (W₁) is reported. The specimens are submerged in water for 24 h at room temperature. After immersion, remove surface moisture and measure saturated weight (W₂). The water absorption percentage is computed using Eq. (1).

(1) $$\text{Absorption}\;\left(\%\right)=\frac{W2-W1}{W1}x\;100$$

#### RCPT

RCPT measures concrete's resistance to chloride ion penetration, which is critical for avoiding reinforcement corrosion. A 50 mm thick, 100 mm diameter concrete disk is formed. The specimen is put between two compartments, one filled with 0.3 N NaOH and the other with 3 % NaCl. A 60 V DC voltage is delivered to the specimen for six hours. The total charge passed (Coulombs) is measured, and lower values indicate superior resistance to chloride penetration.

#### Sulfuric acid resistance test

This test evaluates concrete's ability to withstand acidic environments. Concrete specimens are cured for 28 days. The initial weight (W₀) is recorded. Specimens are immersed in 5 % H₂SO₄ solution for 30 days. The weight is measured periodically to determine deterioration. Acid resistance is determined by weight loss percentage is calculated using the formula (2)

(2) $$\text{Weight}\;\text{loss}\left(\%\right)=\frac{W0-\text{Wt}}{W0}x\;100$$

A lower weight loss percentage indicates better resistance to acid attack.

#### Permeability test

The permeability test determines concrete’s ability to resist water penetration under pressure. A 100 mm diameter and 200 mm height concrete specimen is subjected to water pressure in a permeability cell. Pressure is applied incrementally (e.g., 0.5 MPa to 1.5 MPa) for 24 to 72 h. The penetration depth is measured, and permeability is determined using Darcy’s Law.

#### Sorptivity test

Sorptivity measures the capillary absorption rate of concrete. A 100 mm diameter and 50 mm thick specimen is oven-dried. The specimen’s bottom surface is placed in 500 mm deep water while preventing lateral absorption. The weight is recorded at intervals (5, 10, 30, 60 min). Sorptivity (S) is calculated using Eq. (3)

(3) $$S=\frac{I}{t^{\wedge }0.5}$$

#### Ultrasonic pulse velocity (UPV) test

UPV assesses the quality and uniformity of concrete by measuring the velocity of ultrasonic waves passing through it. The transmitting and receiving transducers are placed on opposite sides of the concrete specimen. A high-frequency pulse is sent through the concrete, and the time taken to pass through is recorded. The velocity (V) is calculated using Eq. (4)

(4) $$V=\frac{L}{T}$$

**Fig. 4:**
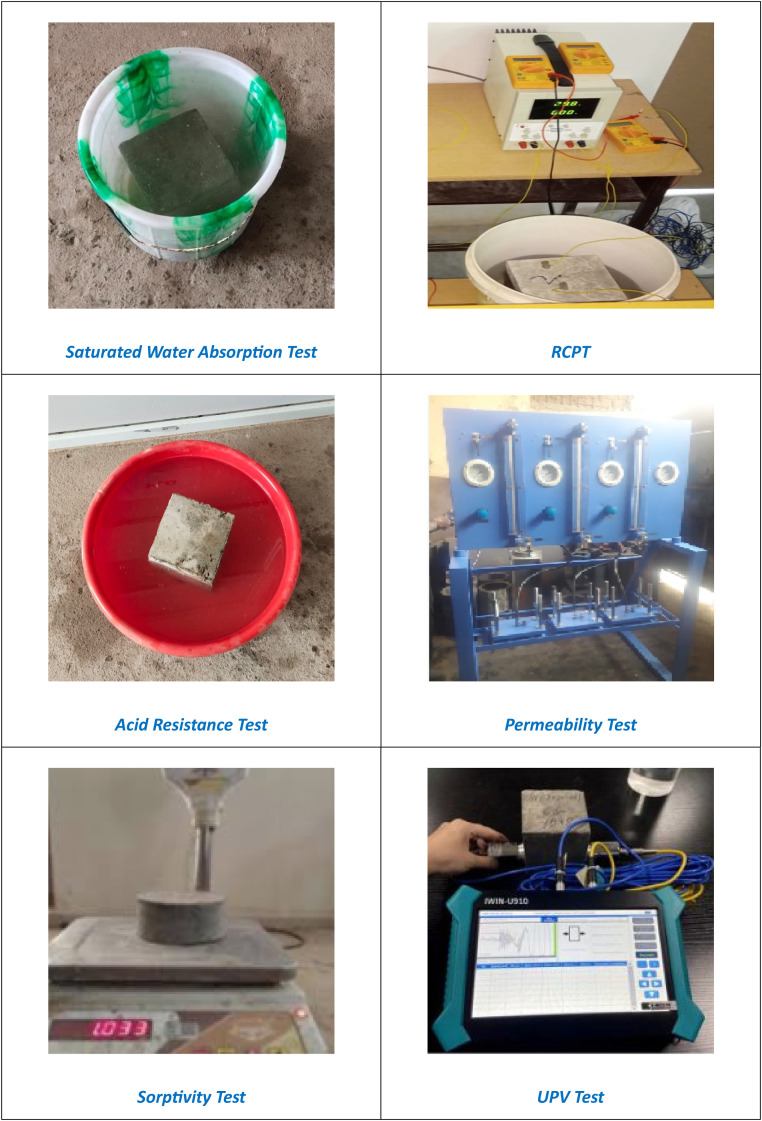
shows the experimental setup of various tests.

Fig. 4 shows the experimental setup of various tests.

## Method validation

### Workability test

This study investigates the effects of partial replacement of cement with flyash and metakaolin, along with the addition of Prosopis Juliflora (P. Juliflora) extract at 50 ppm, on the workability of concrete, as measured by slump value and compaction factor.

Slump value is a measure of the consistency and workability of fresh concrete. The reference mix (M1), with no mineral admixtures or P. Juliflora, recorded a slump of 107 mm. The inclusion of metakaolin led to an initial increase in slump (M2 = 112 mm for 5 %) followed by a decrease with higher replacement levels. M4 (15 % metakaolin) dropped to 102 mm, indicating that excessive metakaolin reduces workability due to its high fineness and pozzolanic activity, which increases water demand. Flyash improved workability compared to metakaolin at similar replacement levels. For instance, M5 and M9 (5 % and 10 % flyash) both maintained slump around 107–111 mm, similar to the control mix, while M13 (15 % flyash) was at 102 mm, showing a mild reduction. This behavior is attributed to the spherical shape and low water demand of flyash. Increasing both admixtures progressively reduced slump values. M16, with 15 % flyash and 15 % metakaolin, recorded the lowest slump (88 mm), indicating significantly reduced workability at higher levels of replacement. The sharp decline is due to the combined high surface area and water demand of both materials.

The compaction factor indicates the degree of workability and is especially useful for low-workability mixes. The control mix (M1) had a compaction factor of 0.892, representing medium workability. Similar to the slump test, a peak compaction factor (0.933) was seen at 5 % metakaolin (M2), after which it decreased with higher replacement (M4 = 0.850). Displayed moderate values (∼0.892 to 0.925), indicating good workability, supporting the spherical particle effect of flyash aiding flowability. As the proportions of both flyash and metakaolin increased, the compaction factor decreased steadily, reaching the lowest value of 0.733 in M16, indicating significantly reduced workability.

**Fig. 5:**
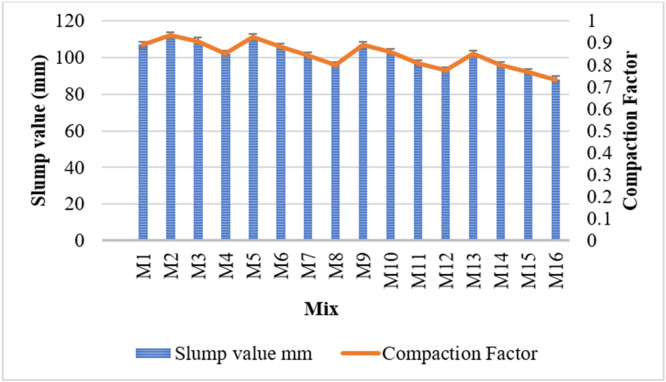
Workability test results.

Low dosages (5 %) of metakaolin or flyash improve workability, likely due to filler effects and improved particle packing. Higher metakaolin content (>10 %) reduces workability more than flyash due to its higher surface area and reactivity. The addition of P. Juliflora (50 ppm) in all mixes may have a slight retarding effect, but its impact on workability appears subtle and dominated by the mineral admixture proportions. Combination of both admixtures at higher dosages severely impairs workability, as evident in M16, necessitating the use of superplasticizers or water adjustments for practical application. Fig. 5 shows the workability test results.

### Compressive strength test

The compressive strength of concrete was evaluated at 7, 14, 28, 56, and 90 days for all 16 mixes containing various proportions of flyash, metakaolin, and a constant 50 ppm dosage of P. Juliflora extract. Mix M1 served as the control, containing only ordinary Portland cement without any admixtures.

Mixes containing only metakaolin (M2 to M4) showed a steady increase in compressive strength as the metakaolin content increased from 5 % to 15 %. At early curing ages (7 and 14 days), these mixes already surpassed the control mix, indicating that metakaolin contributes significantly to early strength gain. This is due to its high pozzolanic reactivity and filler effect, which improve microstructure and hydration. At 90 days, the mix with 15 % metakaolin (M4) achieved a compressive strength of 58.84 MPa, which is significantly higher than the 54.70 MPa recorded for the control mix.

When flyash was used as the sole mineral admixture (in M5, M9, and M13), compressive strength results were slightly improved over the control at all curing periods, particularly at later ages. The early strength gain was modest, as expected, since flyash reacts more slowly than metakaolin. However, by 90 days, mixes with 10–15 % flyash reached strengths of around 58–59 MPa, confirming that flyash contributes more to long-term strength. This aligns with the known pozzolanic activity of flyash, which becomes more pronounced with time.

Mixes containing both flyash and metakaolin showed the most significant improvements in compressive strength, particularly when both materials were used in moderate amounts. Among these, Mix M11, which contained 10 % flyash and 10 % metakaolin, exhibited the highest compressive strength at all ages, culminating in 61.05 MPa at 90 days. This is about 11.6 % higher than the control, showing that combining flyash and metakaolin in equal moderate proportions yields a synergistic effect. The metakaolin accelerates early strength, while flyash enhances strength at later ages.

However, mixes with higher total replacement levels—such as M16, with 15 % of both flyash and metakaolin—showed a decline in strength compared to the optimum combination. While still higher than the control, the 90-day strength of M16 was 56.22 MPa, which is lower than that of M11. This suggests that replacing too much cement can negatively impact strength due to reduced clinker content and insufficient calcium hydroxide to sustain pozzolanic reactions.

Across all mixes, compressive strength increased steadily over time. On average, there was about a 14–15 % increase in strength from 28 to 90 days. This trend was consistent across all mixes, indicating that pozzolanic reactions continue to contribute to strength beyond 28 days, particularly in mixes with flyash. The steady gain confirms the long-term benefits of incorporating supplementary cementitious materials.

The presence of 50 ppm P. Juliflora extract in all modified mixes did not negatively affect compressive strength development. In fact, its consistent inclusion alongside flyash and metakaolin helped achieve higher strength values than the control, suggesting a possible synergistic or neutral effect on hydration and strength development. Further studies would be needed to isolate its chemical influence, but its performance in this study appears beneficial or at least non-detrimental.

**Fig. 6:**
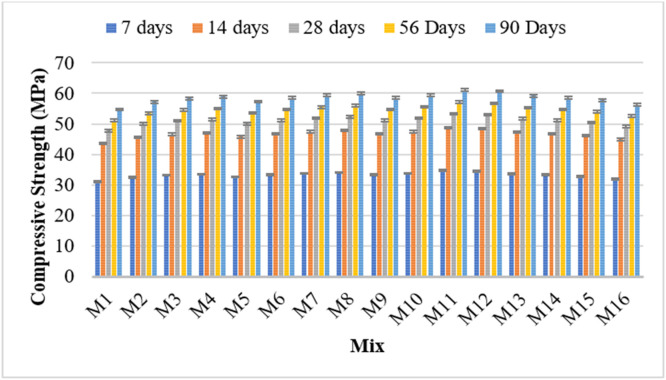
Compressive strength test results.

Metakaolin significantly enhances early and long-term strength, especially at 10–15 % replacement levels. Flyash improves long-term strength but contributes less at early stages. The best performance was observed in Mix M11 (10 % flyash + 10 % metakaolin), indicating an optimal balance of early and late-age strength. Excessive replacement (as in M16) leads to reduced strength, highlighting the need for optimal proportions. P. Juliflora extract at 50 ppm does not hinder strength development and may offer subtle benefits when combined with pozzolanic materials. Fig. 6 shows the compressive strength test results.

### Saturated water absorption test

The SWA Test was conducted to evaluate the porosity and permeability of different concrete mixes over curing periods of 28, 56, and 90 days. The test results indicate a decreasing trend in water absorption as the curing age increases, signifying improved densification and reduced porosity over time.

In the control mix (T1), the water absorption values were 2.82 %, 2.74 %, and 2.65 % for 28, 56, and 90 days, respectively. As metakaolin (MK) and fly ash (FA) were incorporated into the mix, a noticeable reduction in water absorption was observed. This improvement is attributed to the pozzolanic reaction of metakaolin and fly ash, which refines the pore structure and reduces permeability. Mixes containing only metakaolin (T2–T4) showed a gradual reduction in water absorption with increasing metakaolin content. The lowest absorption value in this category was recorded for T4 (15 % MK) at 2.46 % on the 90th day, demonstrating enhanced durability due to the filler effect of metakaolin particles. Similarly, mixes with only fly ash (T5, T9, and T13) exhibited reduced absorption, with T13 (15 % FA) achieving 2.45 % on the 90th day.

**Fig. 7:**
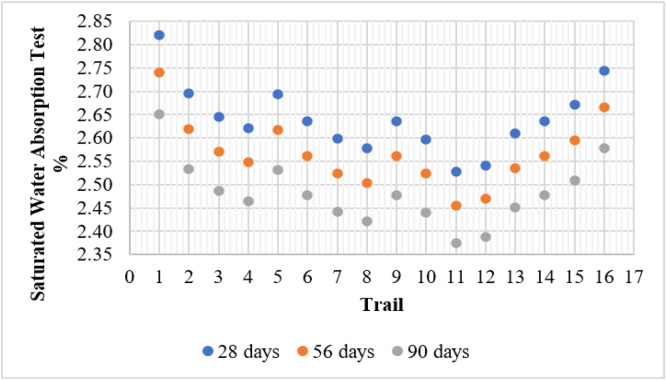
Water Absorption test results.

The combined use of fly ash and metakaolin further improved the results. Among all the mixes, T11 (10 % FA + 10 % MK) had the lowest water absorption at 2.37 % on the 90th day, indicating the optimal balance between pozzolanic activity and microstructure densification. Conversely, mix T16 (15 % FA + 15 % MK) exhibited a slight increase in water absorption compared to other blended mixes, likely due to excessive replacement levels affecting particle packing efficiency. Overall, the incorporation of fly ash and metakaolin significantly reduces water absorption, improving concrete durability by minimizing porosity and enhancing long-term performance. Fig. 7 shows the test results of SWA test.

### RCPT

The RCPT evaluates the resistance of concrete to chloride ion penetration, which is a key factor in assessing durability and corrosion resistance of reinforced structures. The results show a significant reduction in charge passed (Coulombs) with increasing curing age, indicating improved chloride resistance over time.

The control mix (T1) exhibited the highest charge passed values of 3183, 2637, and 2478 Coulombs at 28, 56, and 90 days, respectively. As metakaolin (MK) and fly ash (FA) were incorporated, the charge passed values decreased, signifying enhanced resistance to chloride penetration due to refined pore structure and improved densification. Mixes containing only metakaolin (T2–T4) demonstrated a clear reduction in charge passed with increasing metakaolin content. The lowest value within this category was recorded for T4 (15 % MK) at 2170 Coulombs on the 90th day, reflecting the pozzolanic effect and the formation of a denser microstructure. Similarly, mixes with only fly ash (T5, T9, and T13) exhibited improved performance, with T13 (15 % FA) achieving 2149 Coulombs on the 90th day.

**Fig. 8:**
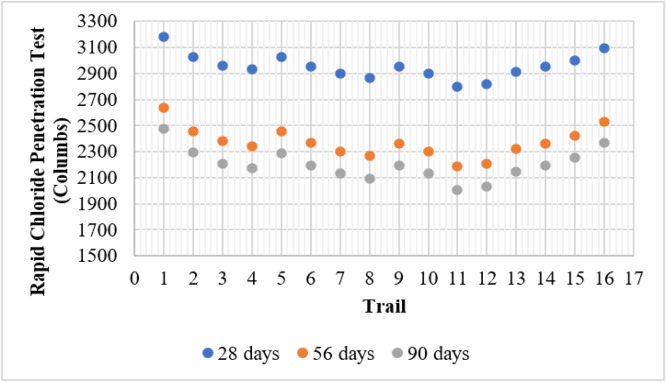
RCPT test results.

Blended mixes containing both fly ash and metakaolin performed even better. Among all the mixes, T11 (10 % FA + 10 % MK) showed the lowest charge passed value of 2007 Coulombs at 90 days, highlighting the synergistic effect of both materials in reducing permeability and enhancing durability. However, mix T16 (15 % FA + 15 % MK) showed a slight increase in charge passed (2365 Coulombs at 90 days), indicating that excessive replacement might affect optimal particle packing and hydration reactions. Overall, the results confirm that incorporating fly ash and metakaolin significantly reduces chloride penetration, thereby improving the durability and service life of concrete structures. Fig. 8 shows the test results of RCPT.

### Acid resistance test

The Sulfuric Acid Resistance Test evaluates the durability of concrete by measuring the percentage weight loss when exposed to acidic environments over 28, 56, and 90 days. The results indicate that weight loss decreases with increasing curing time, demonstrating improved acid resistance with prolonged hydration.

The control mix (T1) recorded the highest weight loss at 7.33 %, 6.30 %, and 6.00 % for 28, 56, and 90 days, respectively. This suggests higher susceptibility to acid attack due to the presence of calcium hydroxide, which reacts with sulfuric acid to form gypsum, leading to concrete deterioration. Mixes containing only metakaolin (T2–T4) exhibited improved acid resistance, with weight loss decreasing as metakaolin content increased. The best performance was observed in T4 (15 % MK), which had a weight loss of 5.55 % at 90 days, demonstrating the pozzolanic effect of metakaolin in reducing calcium hydroxide content and refining the microstructure.

Similarly, mixes with only fly ash (T5, T9, and T13) showed improved resistance compared to the control mix, with T13 (15 % FA) achieving 5.52 % weight loss at 90 days. Fly ash enhances durability by filling voids and forming additional C-S-H gel, reducing acid penetration. The combination of fly ash and metakaolin produced the most acid-resistant concrete. Among all the mixes, T11 (10 % FA + 10 % MK) exhibited the lowest weight loss of 5.31 % at 90 days, confirming the synergistic effect of both materials in reducing porosity and enhancing chemical resistance. However, T16 (15 % FA + 15 % MK) showed slightly higher weight loss (5.83 % at 90 days), likely due to excessive replacement, affecting particle packing efficiency. Fig. 6 shows the weight loss in acid resistance test.

The Sulfuric Acid Resistance Test also assessed the percentage loss in strength of concrete specimens after exposure to an acidic environment for 28, 56, and 90 days. The results indicate that mixes containing fly ash and metakaolin exhibited improved resistance to strength deterioration, with reduced strength loss over time. The control mix (T1) experienced the highest strength loss, with values of 8.27 %, 7.11 %, and 6.77 % at 28, 56, and 90 days, respectively. This can be attributed to the reaction of calcium hydroxide with sulfuric acid, forming expansive and weak gypsum and ettringite phases, leading to deterioration of concrete integrity.

Mixes incorporating only metakaolin (T2–T4) showed reduced strength loss as metakaolin content increased. The best performance was observed in T4 (15 % MK), which recorded a 6.25 % strength loss at 90 days, demonstrating the ability of metakaolin to refine the microstructure and reduce the presence of calcium hydroxide. Similarly, mixes with only fly ash (T5, T9, and T13) exhibited better resistance to strength loss compared to the control mix. The most effective fly ash-based mix, T13 (15 % FA), showed a strength loss of 6.22 % at 90 days, confirming the pozzolanic activity of fly ash in reducing pore connectivity and increasing sulfate resistance.

**Fig. 9:**
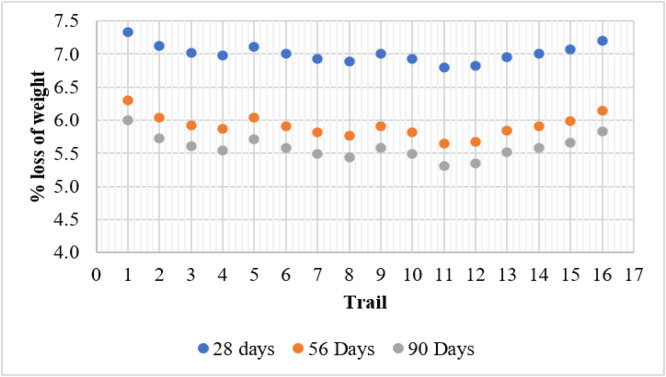
Weight loss in acid resistance test.

**Fig. 10:**
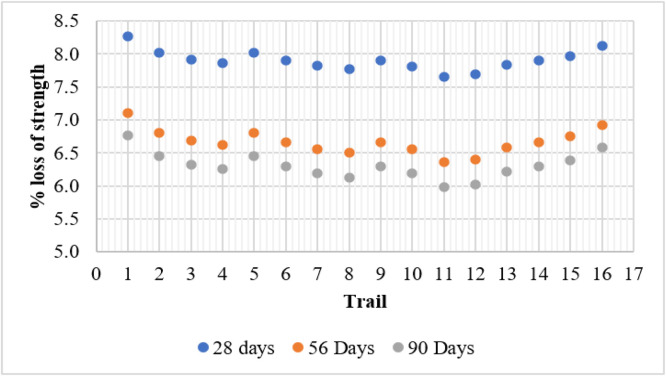
Strength loss in acid resistance test.

The combination of fly ash and metakaolin resulted in the lowest strength loss. The best-performing mix, T11 (10 % FA + 10 % MK), recorded the lowest strength loss of 5.98 % at 90 days, indicating a significant improvement in acid resistance due to the synergistic effect of both pozzolanic materials. However, T16 (15 % FA + 15 % MK) exhibited a slightly higher strength loss (6.58 % at 90 days), likely due to excessive replacement, which may have influenced hydration and strength development. Overall, the results confirm that incorporating fly ash and metakaolin significantly enhances resistance to strength loss in acidic environments, thereby improving the durability and longevity of concrete structures exposed to aggressive conditions. Fig. 9 shows the strength loss in acid resistance test (Fig. 10).

### Permeability test

The Permeability Test evaluates the ability of concrete to resist water penetration, which directly influences its durability. The results indicate that permeability decreases over time across all mixes, confirming that continued hydration and pozzolanic reactions contribute to improved microstructural densification. The control mix (T1) recorded the highest permeability values of 1.84, 1.70, and 1.52 at 28, 56, and 90 days, respectively. This suggests a more porous structure with increased susceptibility to water ingress, which can lead to reduced durability in aggressive environments.

Mixes containing only metakaolin (T2–T4) showed significant improvements in permeability reduction. The best-performing mix in this category, T4 (15 % MK), achieved a permeability of 1.40 at 90 days, demonstrating the refinement of the concrete matrix and reduced pore connectivity due to the pozzolanic reaction of metakaolin. Similarly, mixes with only fly ash (T5, T9, and T13) exhibited better resistance to permeability compared to the control mix. T13 (15 % FA) recorded a permeability of 1.40 at 90 days, showing that fly ash contributes to densification by forming additional C-S-H gel, which fills voids and reduces water penetration.

**Fig. 11:**
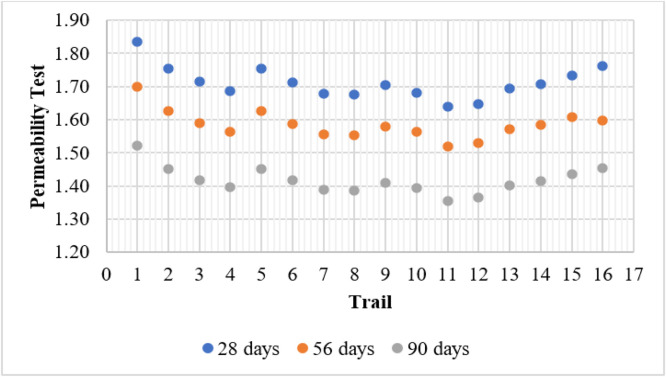
Permeability test results.

The combination of fly ash and metakaolin further improved permeability resistance. Among all mixes, T11 (10 % FA + 10 % MK) exhibited the lowest permeability of 1.36 at 90 days, highlighting the synergistic effect of these pozzolanic materials in refining the microstructure and reducing porosity. However, T16 (15 % FA + 15 % MK) exhibited slightly higher permeability (1.45 at 90 days), which may be due to excessive replacement leading to changes in particle packing efficiency. Overall, the results confirm that incorporating fly ash and metakaolin significantly enhances concrete's impermeability, thereby improving its resistance to moisture penetration and prolonging structural durability in harsh conditions. Fig. 11 shows the permeability test results.

### Sorptivity test

The sorptivity test measures the rate of capillary water absorption in concrete, which directly affects its durability. Lower sorptivity values indicate improved resistance to moisture ingress, reducing the risk of deterioration caused by water-related damage. The control mix (T1) exhibited the highest sorptivity values of 0.086, 0.082, and 0.074 at 28, 56, and 90 days, respectively. This suggests that concrete without supplementary cementitious materials (SCMs) has a relatively porous microstructure, allowing higher water absorption.

Mixes incorporating only metakaolin (T2–T4) showed a noticeable reduction in sorptivity. The best-performing mix in this category, T4 (15 % MK), recorded sorptivity values of 0.080, 0.076, and 0.069 at 28, 56, and 90 days, respectively. This reduction can be attributed to metakaolin’s high reactivity, which refines the concrete’s microstructure by enhancing the C-S-H gel formation and reducing pore sizes. Similarly, mixes containing only fly ash (T5, T9, and T13) demonstrated improved water absorption resistance compared to the control mix. T13 (15 % FA) exhibited a sorptivity value of 0.068 at 90 days, indicating that fly ash effectively enhances the densification of concrete over time due to its delayed pozzolanic reaction.

**Fig. 12:**
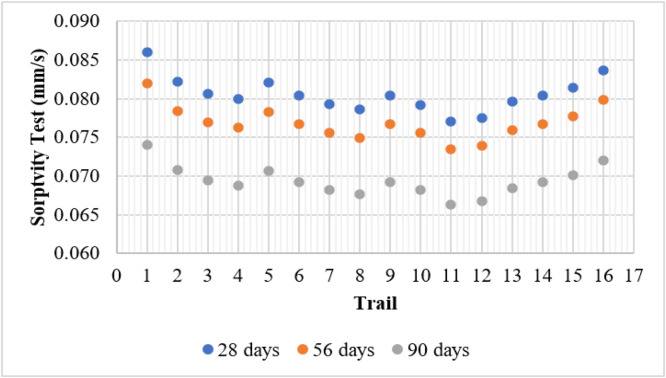
Sorptivity test results.

The combination of fly ash and metakaolin provided the most significant reduction in sorptivity. T11 (10 % FA + 10 % MK) recorded the lowest sorptivity value of 0.066 at 90 days, highlighting the synergistic effect of these materials in reducing capillary pores and enhancing water resistance. However, T16 (15 % FA + 15 % MK) showed a slight increase in sorptivity (0.072 at 90 days), possibly due to excessive replacement affecting the particle packing efficiency. Overall, the results confirm that incorporating fly ash and metakaolin in optimal proportions significantly reduces water absorption, thereby improving the durability and longevity of concrete structures in moisture-prone environments. Fig. 12 shows the sorptivity test results.

### UPV test

The Ultrasonic Pulse Velocity (UPV) test determines the quality and integrity of concrete by measuring the speed of ultrasonic waves passing through the material. Higher UPV values imply a denser and more homogeneous concrete matrix, contributing to improved durability and mechanical performance. The control mix (T1) exhibited UPV values of 3790 m/s, 4282 m/s, and 4387 m/s at 28, 56, and 90 days, respectively. This indicates that conventional concrete without supplementary cementitious materials maintains a relatively strong internal structure but may be prone to gradual deterioration under aggressive conditions.

The incorporation of only metakaolin (T2–T4) led to slight increases in UPV compared to the control mix. The lowest velocity in this group was seen in T2 (5 % MK) with 3865 m/s, 4335 m/s, and 4436 m/s, while T4 (15 % MK) reached 3910 m/s, 4368 m/s, and 4465 m/s at 28, 56, and 90 days, respectively. These results reflect metakaolin’s ability to refine pore structure and contribute to pozzolanic reactions, enhancing long-term strength. Similarly, fly ash-only mixes (T5, T9, T13) showed progressive improvement in UPV over time. T5 (5 % FA) recorded 3867 m/s, 4337 m/s, and 4437 m/s, while T13 (15 % FA) achieved 3918 m/s, 4373 m/s, and 4470 m/s. Although fly ash reacts more slowly than metakaolin, its contribution to long-term densification becomes evident with extended curing.

The combined use of fly ash and metakaolin showed even more promising results. T11 (10 % FA + 10 % MK) displayed the highest UPV values among the ternary blends: 3967 m/s, 4408 m/s, and 4502 m/s. This indicates that the synergy between the two materials promotes effective microstructural development and pore refinement. T16 (15 % FA + 15 % MK) also performed well, recording UPVs of 3836 m/s, 4315 m/s, and 4417 m/s, suggesting balanced hydration and packing density.

**Fig. 13:**
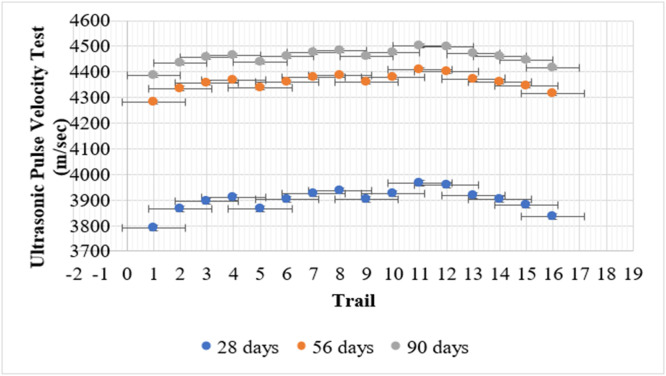
shows the UPV test results.

Overall, the UPV results show that incorporating P. juliflora extract with optimized fly ash and metakaolin blends enhances long-term concrete quality. Despite initial variations in UPV, the improved durability indicators—such as permeability and RCPT—confirm the beneficial effects of these supplementary materials in refining the microstructure and boosting performance over time. Fig. 13 shows the UPV test results.

## Statical analysis

### Compressive strength test

A one-way ANOVA was performed to assess the differences in compressive strength among five groups of concrete samples, each corresponding to a different curing age (7, 14, 28, 56, and 90 days), with 16 observations per group. The mean compressive strengths for these groups were 33.24, 46.67, 51.06, 54.64, and 58.46 MPa, respectively, indicating a consistent increase in strength over time. The ANOVA results showed a between-group sum of squares of 6066.49 and a within-group sum of squares of 138.56. The calculated F-value was 820.92, which is substantially higher than the F-critical value of 2.49. Additionally, the P-value was 4.56 × 10⁻⁶¹, strongly confirming that the differences among the groups are statistically significant. These results demonstrate that curing duration has a significant effect on the compressive strength of concrete mixes containing flyash, metakaolin, and P. Juliflora extract. The increasing trend in strength over time suggests continued pozzolanic activity and densification of the microstructure, contributing to improved mechanical performance. Overall, the analysis confirms that extended curing significantly enhances the strength development of modified concrete mixes, which is crucial for structural applications requiring long-term durability. Table 4 shows the single factor ANOVA for compressive strength test results.

**Table 4:** Single factor ANOVA for compressive strength.

Source of Variation	SS	df	MS	F	P-value	F crit
Between Groups	6066.493	4	1516.623	820.9231	4.56E-61	2.493696
Within Groups	138.5596	75	1.847461			
Total	6205.052	79				

### RCPT

A one-way ANOVA was performed to assess the differences in Rapid Chloride Penetration Test (RCPT) results among three groups of concrete samples, each consisting of 16 observations. The mean RCPT values for 28, 56 and 90 days were 2952.74, 2368.36, and 2198.48, respectively, indicating a progressive reduction in chloride ion permeability. The ANOVA results showed a between-group sum of squares of 5009,373.37 and a within-group sum of squares of 553,580.11. The computed F-value was 203.60, which is substantially higher than the F-critical value of 3.204. Additionally, the P-value was 2.83 × 10⁻²³, confirming that the differences among the groups are statistically significant. These findings indicate that the treatments or modifications applied to the concrete mixes significantly influenced their resistance to chloride ion penetration. The decreasing trend in RCPT values suggests that each subsequent modification led to improved durability performance, likely due to enhancements in pore structure or the use of supplementary materials. Overall, the analysis confirms that the tested interventions effectively improved the concrete’s resistance to chloride ingress, which is critical for long-term structural durability in aggressive environments. Table 5 shows the single factor ANOVA for RCPT results.

**Table 5:** Single factor ANOVA for RCPT.

Source of Variation	SS	df	MS	F	P-value	F crit
Between Groups	5009,373	2	2504,687	203.6036	2.83E-23	3.204317
Within Groups	553,580.1	45	12,301.78			
Total	5562,953	47				

### Permeability test

A one-way ANOVA was conducted to evaluate the differences in permeability test results among three groups of concrete samples, each containing 16 observations. The mean permeability values were 7.91 for 28 days, 6.66 for 56 days, and 6.30 for 90 days, showing a consistent decline across the groups. The ANOVA results revealed a between-group sum of squares of 22.66 and a within-group sum of squares of 1.48. The resulting F-value of 345.39 far exceeds the critical value of 3.204, and the P-value of 4.96 × 10⁻²⁸ indicates a statistically significant difference among the groups. This strong significance suggests that the variations in permeability are not due to random chance, but rather due to changes in the concrete mix or treatment applied. The decreasing trend in average permeability values implies improved resistance to water penetration, potentially due to a denser matrix, reduced porosity, or the inclusion of permeability-reducing admixtures. These results confirm that the modifications made to the concrete significantly enhance its impermeability, which is critical for durability in moisture-sensitive environments. Table 6 shows the single factor ANOVA for permeability results.

**Table 6:** Single factor ANOVA for permeability.

Source of Variation	SS	df	MS	F	P-value	F crit
Between Groups	22.65865	2	11.32932	345.3858	4.96E-28	3.204317
Within Groups	1.476087	45	0.032802			
Total	24.13474	47				

## Conclusion

The experimental investigation on the effects of fly ash, metakaolin, and P. juliflora extract on concrete durability and performance demonstrated significant improvements in key properties. The optimal concrete mix was found with 10 % flyash and 10 % metakaolin, showing maximum compressive strength. Metakaolin enhanced early strength, while flyash contributed to long-term gains. Excessive replacement reduced performance. P. Juliflora at 50 ppm had no adverse effects, supporting its compatibility with pozzolanic materials in concrete.

The Sulphuric Acid Resistance Test demonstrated that the weight and strength loss due to acid exposure was lower in mixes containing metakaolin and fly ash, highlighting their role in increasing acid resistance. Similarly, the Permeability Test showed a reduction in permeability with increasing fly ash and metakaolin content, indicating improved microstructural refinement. The Sorptivity Test results reinforced this observation, with lower absorption rates in modified mixes, confirming reduced capillary action and improved water resistance.

The UPV Test results revealed that while the incorporation of supplementary cementitious materials initially reduced wave propagation speed, the long-term densification effect improved concrete homogeneity and quality. Among the various combinations, mixes with 10–15 % fly ash and 10–15 % metakaolin exhibited the best overall performance across all tests, proving that the optimal blend of these materials enhances durability and mechanical properties.

In conclusion, the use of fly ash, metakaolin, and P. juliflora extract in concrete significantly enhances resistance to water absorption, chloride penetration, acid attack, and permeability while maintaining structural integrity. This research supports the sustainable use of industrial by-products and natural plant extracts to develop durable and eco-friendly concrete, making it a viable alternative for long-lasting infrastructure applications.

## Limitations

Not applicable.

## Ethical statements

The paper reflects the authors' own research and analysis in a truthful and complete manner.

## CRediT authorship contribution statement

**Sajeev P S:** Conceptualization, Methodology, Writing – original draft. **Vijay Shankar Giri Rajagopal:** Conceptualization, Supervision, Writing – review & editing. **Naveen Arasu A:** Resources, Supervision, Project administration, Writing – review & editing.

## Declaration of competing interest

The authors declare that they have no known competing financial interests or personal relationships that could have appeared to influence the work reported in this paper.

## Data Availability

Data will be made available on request.
